# Prediction models for patients with esophageal or gastric cancer: A systematic review and meta-analysis

**DOI:** 10.1371/journal.pone.0192310

**Published:** 2018-02-08

**Authors:** H. G. van den Boorn, E. G. Engelhardt, J. van Kleef, M. A. G. Sprangers, M. G. H. van Oijen, A. Abu-Hanna, A. H. Zwinderman, V. M. H. Coupé, H. W. M. van Laarhoven

**Affiliations:** 1 Cancer Center Amsterdam, Amsterdam, The Netherlands; 2 Department of Medical Oncology, Academic Medical Center, University of Amsterdam, Amsterdam, The Netherlands; 3 Amsterdam Public Health Research Institute, Amsterdam, The Netherlands; 4 Department of Epidemiology and Biostatistics, VU University Medical Center, Amsterdam, The Netherlands; 5 Department of Medical Psychology, Academic Medical Center, University of Amsterdam, Amsterdam, The Netherlands; 6 Department of Medical Informatics, Academic Medical Center, University of Amsterdam, Amsterdam, The Netherlands; 7 Department of Clinical Epidemiology, Biostatistics and Bioinformatics, Academic Medical Center, University of Amsterdam, Amsterdam, The Netherlands; National Cancer Center, JAPAN

## Abstract

**Background:**

Clinical prediction models are increasingly used to predict outcomes such as survival in cancer patients. The aim of this study was threefold. First, to perform a systematic review to identify available clinical prediction models for patients with esophageal and/or gastric cancer. Second, to evaluate sources of bias in the included studies. Third, to investigate the predictive performance of the prediction models using meta-analysis.

**Methods:**

MEDLINE, EMBASE, PsycINFO, CINAHL, and The Cochrane Library were searched for publications from the year 2000 onwards. Studies describing models predicting survival, adverse events and/or health-related quality of life (HRQoL) for esophageal or gastric cancer patients were included. Potential sources of bias were assessed and a meta-analysis, pooled per prediction model, was performed on the discriminative abilities (c-indices).

**Results:**

A total of 61 studies were included (45 development and 16 validation studies), describing 47 prediction models. Most models predicted survival after a curative resection. Nearly 75% of the studies exhibited bias in at least 3 areas and model calibration was rarely reported. The meta-analysis showed that the averaged c-index of the models is fair (0.75) and ranges from 0.65 to 0.85.

**Conclusion:**

Most available prediction models only focus on survival after a curative resection, which is only relevant to a limited patient population. Few models predicted adverse events after resection, and none focused on patient’s HRQoL, despite its relevance. Generally, the quality of reporting is poor and external model validation is limited. We conclude that there is a need for prediction models that better meet patients’ information needs, and provide information on both the benefits and harms of the various treatment options in terms of survival, adverse events and HRQoL.

## Introduction

Worldwide, esophageal and gastric cancer account for 3.2% and 6.8% of all new cancer cases, respectively. The prognosis is dismal: 1% of patients with esophageal cancer and 5% of patients with gastric cancer survive at least 5 years after being diagnosed[1]. However, survival rates for both entities vary greatly[1–4] and metastasis is one of the decisive factors for curative or palliative treatment. In both the curative and palliative setting, patients may choose between various treatment options that differ in terms of efficacy, adverse events and impact on health-related quality of life (HRQoL).

Many patients with potentially curable esophageal or gastric cancer report loss of HRQoL[5, 6] during the first year after surgery, even though patients indicate that an improved HRQoL may be their primary outcome of treatment[7]. Likewise, one in four patients with metastatic esophageal cancer state that HRQoL is their main treatment goal[8]. Since life prolonging treatment may come at a cost as it may induce adverse events and impair HRQoL[5, 6], patients need to be informed at an early stage about the projected survival, adverse events and HRQoL.

To make well-informed treatment choices that match patients’ preferences and goals, information about treatment outcomes in terms of survival, treatment-related adverse events and HRQoL is necessary[9]. Statistical prediction models that provide personalized estimates of such outcomes can help inform patients and clinicians consequently supporting shared decision-making. Such statistical models are generally derived from large historical patient cohorts. Examples of such models in oncology are Adjuvant![10] and PREDICT[11], which are broadly used in the field of breast cancer. However, a comprehensive overview of available models for esophageal and gastric cancer, and their predictive performance is currently lacking. Therefore, the aim of this systematic review was first to provide an overview of published prediction models that provide personalized estimates of survival probabilities (i.e., overall, disease-specific, progression-free or disease-free survival), the probability of developing treatment-related adverse events, and/or the impact of treatment on HRQoL. Secondly, we aimed to examine the quality of the development and validation studies conducted for the identified prediction models. Finally, we evaluated the reported performance of the prediction models in terms of discriminative ability and calibration.

## Methods

### Systematic literature search

A systematic literature search was performed to identify all relevant publications in the bibliographic databases MEDLINE, EMBASE, PsycINFO, CINAHL, and The Cochrane Library (no protocol available). To increase the relevance of the findings of this review for current clinical practice, we only included papers published from January, 1^st^ 2000 up to February 6^th^, 2017. Search terms for ‘esophageal cancer’ or ‘gastric cancer’ were used in combination with search terms for ‘prediction model’, ‘survival’, ‘adverse events’ and ‘quality of life’ (see S1 Table for the detailed search strategy). The reference list of relevant articles identified were also searched for additional relevant publications.

The aim of our search was to identify prediction models that provide personalized estimates of survival, the probability of experiencing an adverse event and/or the impact of disease or treatment on HRQoL for esophageal and gastric cancer patients. Models intended to support treatment decisions in both the curative or the palliative setting were eligible for inclusion. Studies validating models in patients with esophageal or gastric cancer that were not originally developed for use in these populations, were also eligible for inclusion. Also, only papers published in English were assessed. We excluded studies describing prediction models that aimed to classify patients into risk categories (such as “low risk” and “high risk”), rather than providing personalized estimates of outcome probability. Although risk categories may be useful for discriminating between outcome severity, it is difficult to quantify the calibration of such prediction models (i.e., how does the expected outcome compare to the actual observed outcome). This is an important aspect of model validation, as the absolute outcome probabilities are needed to determine model fit, and therefore, the quality of the model.

The selection process consisted of two phases. First, all titles and abstracts were screened by two reviewers (HvdB and EE) independently. Discrepancies were resolved through consensus, and when necessary by consulting a third arbiter (HvL). Studies were also selected if eligibility could not be determined on the basis of the titles and abstracts. In the second phase, two reviewers (HvdB and EE) independently screened full texts of the studies selected in phase 1 to determine eligibility conclusively.

### Data extraction

Data were extracted from the full text papers according to the CHARMS[12] statement, which provides a data extraction checklist for systematic reviews of prediction models. Extracted data included information about the type of article, study design, data source, characteristics of the population, aim of the model, type of outcome, sample size, methods used and presentation of the final prediction model. Model performance was also extracted and categorized as development performance (obtained when using the development dataset), internal validation performance (obtained when using data from a population similar to that of the development set), and external validation performance (when the data used differs temporally, geographically etc. from the development set). Model performance was described using measures for discriminative ability and measures for calibration. Discriminative ability is defined as a model’s ability to differentiate between patients who experience an event (such as death or an adverse event) and those who do not[13]. This can be quantified by calculating an index of predictive discrimination, the concordance index (c-index). This c-index typically has values ranging from 0.5 (no discrimination at all) to 1 (perfect discrimination), and is the generalization of the area under the curve, a well-known measure of discrimination. Typically c-indices can be interpreted by the following rule of thumb: 0.5–0.6 no discrimination, 0.6–0.7 poor, 0.7–0.8 fair, 0.8–0.9 good and 0.9–1 excellent discrimination. Model calibration, in contrast, conveys the goodness of fit, i.e., the congruence between observed and average predicted outcomes[13]. Calibration can be displayed visually in a calibration plot.

The levels of evidence of the discriminatory accuracy of the prediction model as described by Reilly and Evans[14], indicates how extensively a prediction model has been validated and to what extent a model is ready for clinical use. Level 1 refers to model development, level 2 to narrow validation, level 3 to broader validation and level 4 and 5 to respectively narrow or broad impact analysis. Each identified study was categorized according to the Reilly-Evans levels. For the assessment of bias, there are no established checklists specifically designed for use in prediction modelling studies. We therefore created a classification system for several areas of possible bias, which were derived from the TRIPOD-statement (transparent reporting of a multivariable prediction model for individual prognosis or diagnosis)[15]. S2 Table presents an overview of the classification system used for potential risk of bias.

Data extraction was performed by two researchers (HvdB and EE). First, a subset of 10 articles was used as a training set. The training set was coded by both researchers independently and discrepancies in coding were resolved during a consensus meeting. The percentage overall agreement between the two coders was approximately 90% across individual items. The coding scheme was revised where necessary as a result of the training set findings. Thereafter, each researcher coded half of the remaining articles. Classification of the potential for bias was done in two stages; each researcher made notes of potential sources of bias per category separately, and together they (HvdB and EE) then categorized the identified potential sources of bias. The bias was determined in six areas: population-related (such as selection bias), predictor-related (such as ill-defined predictors), outcome-related (such as an unclear outcome), sample size-related, missing data-related (such as only complete case analysis) and statistical analysis-related (such as underreporting of statistics).

### Bias analyses

Descriptive analyses were used to summarize study and model characteristics. We expected that the higher the impact factor of a journal in which the study was published, the more stringent the internal screening and peer review procedures would be and, hence, the lower the risk of bias. Further, we hypothesized that the higher the impact factor of the journal a prediction model was published in, the better its performance in terms of c-index would be. Both hypotheses were assessed through the Spearman rank correlation between the journal impact factor[16] (in the year of publication, or the closest to publication year available) and the reported c-index as well as between journal impact factor and the potential sources of bias (assessed using the classification of potential sources of bias presented in S2 Table), respectively. Due to differences in esophageal carcinoma histology in different geographical populations[17], we examined whether models were constructed and validated with patient cohorts from different continents using the Fisher’s exact test. Finally, we hypothesized that the reported c-indices would be larger during model development than during validation due to overfitting. This was assessed using a one-tailed Wilcoxon signed-rank test. These analyses were performed in the R-studio environment with R version 3.3.3 (R Foundation for Statistical Computing, Vienna, Austria, https://www.r-project.org).

### Meta-analyses of c-indices

To gain insight in the discriminative abilities of the prediction models, we performed meta-analyses. The c-indices were pooled per prediction model using random effects modelling for models for which at least two concordance indices were available. Analyses were performed using linear restricted maximum-likelihood estimation. In most articles, the c-index confidence interval or variance was not reported. In those cases, the study weights in the meta-analysis were determined as the inverse square root of the sample size. The logistic transformation as described in Kottas et al.[18] was applied to all c-index estimates during calculations and then transformed back; this procedure ensures that all estimates are bounded by 0 and 1 after pooling, which is a property of the c-index. These analyses were performed using the Metafor package in the R-studio environment (R version 3.3.3).

## Results

**Fig 1:**
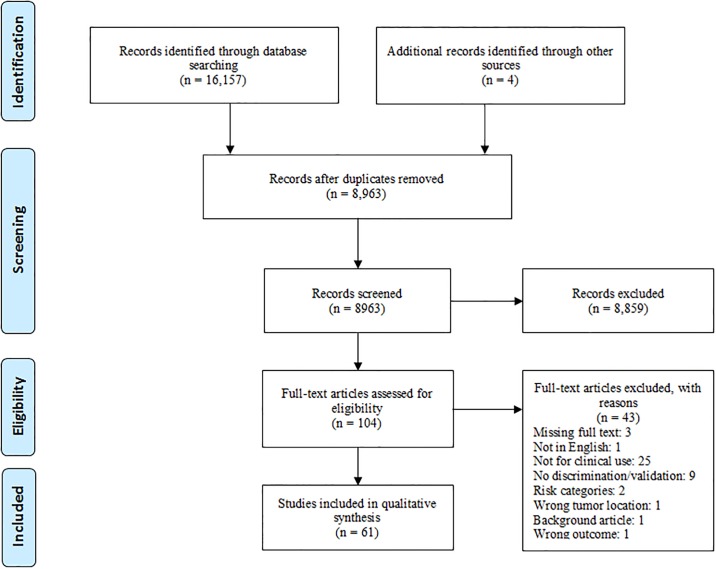
Overview of study selection according to the “Preferred Reporting Items for Systematic Reviews and Meta-Analyses” (PRISMA) statement[21].

A total of 8,963 articles was identified, of which 61 were eligible for inclusion in this systematic review (Fig 1). These studies described a grand total of 47 prediction models for patients with esophageal or gastric cancer. Two studies describing the development of a prediction model, were not included in our systematic review due to the publication year (POSSUM[19]), and incorrect patient population (P-POSSUM[20]). The remaining 45 development studies are shown in Table 1. Further, we found 16 validation studies on a total of 10 prediction models. These studies are shown in Table 2.

**Table 1:** Overview of selected studies which describe the creation of a novel prediction model.

**Study**	N	Country	Tumor location	Treatment intention	Outcome	Model c-indices	Model presentation	Reilly-Evans level
Biglarian 2011[22]	300	Iran	Stomach	Unclear	OS	OS: 0.88 (dev), 0.92 (int)	None	1
Cao 2016[23]	4281	USA, China	Esophagus	Unclear	CSS	DSS: 0.72 (dev), 0.699 (ext)	Nomogram	2
Chen, S. 2016[24]	308	China	Esophagus	Curative	DSS	DSS: 0.688 (dev)	Nomogram	1
Deans 2007[25]	220	UK	Esophagogastric	Curative/Palliative	OS	OS: 0.84 (dev), 0.85 (dev)	Formula	1
Dhir 2012[26]	14235	USA	Stomach	Curative/Palliative	POM	POM: 0.75 (ext)	Nomogram	2
Dikken 2013[27]	1642	USA/NL	Stomach	Curative	DSS	DSS: 0.77 (dev)	Nomogram	1
Duan 2016[28]	328	China	Esophagus	Curative	OS, DFS	OS: 0.71 (dev), 0.77 (int); DFS: 0.71 (dev), 0.65 (int)	Nomogram	1
Eil 2014[29]	824	USA	Esophagus	Unclear	OS	OS: 0.72 (dev)	Online tool	1
Eom 2015[30]	1579	Korea	Stomach	Curative	OS	OS: 0.831 (ext)	Nomogram	3
Filip 2015[31]	167	Italy	Esophagus	Unclear	AE	AE: 0.8 (dev)	Formula	3
Fischer 2016[32]	4882	UK	Esophagogastric	Curative	POM, AE	POM: 0.698 (dev), 0.694 (dev); AE: 0.631 (dev)	Formula	1
Fuccio 2016[33]	267	Italy	Esophagus	Curative/Palliative	AE	AE: 0.617 (dev), 0.617 (dev), 0.622 (dev)	Table	1
Gabriel 2017[34]	7179	USA	Esophagus	Curative	OS	OS: 0.656 (dev), 0.669 (dev), 0.63 (int), 0.682 (int)	Formula	1
Haga 2015[35]	762	Japan	Stomach	Unclear	OS	OS: 0.89 (dev)	Formula	1
Han 2012[36]	5300	Korea, Japan	Stomach	Unclear	OS	OS: 0.78 (int), 0.79 (ext)	Nomogram	2
Hirabayashi 2014[37]	3085	Japan	Stomach	Curative	OS	OS: 0.68 (ext)	Nomogram	2
Jiang 2016[38]	125	China	Stomach	Unclear	OS	OS: 0.868 (int), 0.698 (int), 0.84 (int), 0.786 (int), 0.836 (ext), 0.669 (ext), 0.832 (ext), 0.749 (ext)	Nomogram	2
Jung 2013[39]	239	Korea	Esophagus	Palliative	OS	OS: 0.69 (dev)	Nomogram	1
Kattan 2003 (MSKCC)[40]	1039	USA	Stomach	Curative	DSS	DSS: 0.8 (dev)	Nomogram, online tool	3
Kim, Y. 2015[41]	719	USA	Stomach	Curative	OS, DFS	OS: 0.711 (dev), 0.691 (ext); DFS: 0.702 (dev), 0.685 (ext)	Nomogram	1
Kunisaki 2016[42]	52770	Japan	Stomach	Unclear	AE	AE: 0.797 (int), 0.784 (int), 0.748 (int), 0.832 (int), 0.728 (int), 0.7 (int), 0.779 (int), 0.658 (int)	Formula	2
Kurita 2015[43]	33917	Japan	Stomach	Curative	POM	POM: 0.785 (dev), 0.798 (int)	None	1
Lagarde 2007b[44]	364	Unclear	Esophagus	Curative	DSS	DSS: 0.77 (dev)	Nomogram	2
Lagarde 2008a[45]	663	Netherlands	Esophagus	Curative	AE	AE: 0.65 (dev), 0.666 (int)	Nomogram	3
Lai 2009[46]	2923	Korea	Stomach	Curative	DFS	DFS: 0.79 (dev)	None	2
Liu, J. 2016a[47]	817	China	Stomach	Unclear	OS	OS: 0.79 (ext)	Nomogram	1
Liu, J. 2016b[48]	2770	USA, China	Stomach	Curative	DSS	DSS: 0.73 (int), 0.76 (ext)	Nomogram	2
Liu, J.S. 2015[49]	326	China	Esophagus	Curative	DSS	DSS: 0.72 (dev)	Nomogram	1
Marrelli 2005[50]	536	Italy	Stomach	Curative	DFS	DFS: NA (dev)	Formula	2
Mohammadzadeh 2015[51]	194	Iran	Stomach	Unclear	OS	OS: 0.8 (dev), 0.79 (int)	Decision tree	1
Muneoka 2016[52]	207	Japan	Stomach	Curative	DFS	DFS: 0.8 (dev)	Nomogram, online tool	1
Shao 2015[53]	633	China	Esophagus	Curative	OS	OS: 0.77 (dev), 0.77 (dev), 0.76 (int), 0.77 (int)	Nomogram	1
Shapiro 2016[54]	626	Netherlands	Esophagus	Curative	OS	OS: 0.63 (dev)	Nomogram	1
Shiozaki 2016[55]	64	USA	Esophagogastric	Palliative	OS	OS: 0.61 (dev)	Nomogram	1
Song 2014[56]	805	Korea	Stomach	Curative	DSS	DSS: 0.87 (dev), 0.84 (int)	Nomogram, formula	1
Steyerberg 2006[57]	1327	USA, Netherlands	Esophagus	Unclear	POM	POM: 0.66 (dev), 0.7 (ext), 0.56 (ext), 0.66 (ext)	Formula	3
Su 2015[58]	797	China	Esophagus	Unclear	OS	OS: 0.73 (dev), 0.715 (int)	Nomogram	1
Suzuki 2012[59]	196	USA	Esophagus	Unclear	OS, DFS	OS: 0.7 (dev); DFS: 0.77 (dev)	Nomogram	1
Tekkis 2004 (O-POSSUM)[60]	1042	UK	Esophagogastric	Curative/Palliative	POM	POM: 0.8 (dev)	Formula	3
Tu 2017[61]	3632	China	Stomach	Curative	AE	AE: 0.68 (dev)	Nomogram	1
Woo 2016[62]	11851	Korea, Japan, China	Stomach	Curative/Palliative	OS	OS: 0.824 (dev), 0.842 (ext), 0.868 (ext), 0.839 (ext), 0.798 (ext)	Formula	3
Yang 2013[63]	319	China	Esophagus	Curative	REC	Not available	Formula	1
Yu 2016[64]	1004	China	Esophagus	Curative	OS	OS: 0.7 (dev)	Nomogram	1
Zhao 2016[65]	510	China	Stomach	Curative	OS	OS: 0.834 (dev), 0.809 (int)	Nomogram	1
Zhou, Z. 2015[66]	953	USA, China	Esophagus	Curative	OS	OS: 0.69 (dev), 0.75 (ext)	Nomogram	2

N: sample size used for training. DSS: disease-specific survival, POM: post-operative mortality, OS: overall survival, AE: adverse events, DFS: disease-free survival, REC: cancer recurrence. The type of validation is indicated in brackets with the reported c-index; dev: development c-index, int: internal validation, ext: external validation.

**Table 2:** Overview of studies which externally validate prediction models.

Study	Validation of	N	Country	Tumor location	Treatment intention	outcome	Model C-indices	Reilly-Evans level
Ashfaq 2015[67]	MSKCC[40]	6954	USA	Stomach	Curative	DSS	DSS: 0.68	3
Bosch 2011[68]	P-POSSUM[20], O-POSSUM[60]	278	Netherlands	Esophagus	Curative	POM	POM: 0.766, 0.756	3
Chen, D. 2013[69]	MSKCC[40]	979	China	Stomach	Curative	DSS	DSS: 0.74	3
D’Journo 2016[70]	Steyerberg 2006[57]	1039	France	Esophagus	Unclear	OS	OS: 0.63, 0.64, 0.63	3
Dikken 2014[71]	MSKCC[40]	139	USA	Stomach	Unclear	DSS	DSS: 0.64	3
Grotenhuis 2010[72]	Lagarde 2008a[45]	777	Netherlands	Esophagus	Curative	AE	AE: 0.64	3
Kim, J.H. 2012[73]	Lai 2009[46]	930	Korea	Stomach	Curative	DFS	DFS: 0.7	2
Lagarde 2007a[74]	O-POSSUM[60]	663	Netherlands	Esophagus	Curative	POM	POM: 0.6	3
Lagarde 2008b[75]	Lagarde 2007b[44]	382	Belgium	Esophagus	Curative	DSS	DSS: 0.76	2
Marrelli 2015[76]	Marrelli 2005[50]	635	Italy	Stomach	Curative	REC	REC: 0.889	2
Nagabhushan 2007[77]	P-POSSUM[20], O-POSSUM[60]	313	UK	Esophagogastric	Curative	POM	POM: 0.68, 0.61	3
Novotny 2006[78]	MSKCC[40]	862	Germany	Stomach	Curative	DSS	DSS: 0.77	3
Peeters 2005[79]	MSKCC[40]	459	Netherlands	Stomach	Curative	DSS	DSS: 0.77	3
Reim 2015[80]	Eom 2015[30]	908	Germany	Stomach	Curative	OS	OS: 0.761	3
Zafirellis 2002[81]	POSSUM[19]	204	UK	Esophagus	Curative/Palliative	OS, AE	OS: 0.62; AE: 0.55	3
Zhou, M.L. 2016[82]	MSKCC[40]	150	China	Stomach	Curative	DSS	DSS: 0.657	3

N: sample size used for validation, DSS: disease-specific survival, POM: post-operative mortality, OS: overall survival, AE: adverse events, DFS: disease-free survival, REC: cancer recurrence.

**Fig 2:**
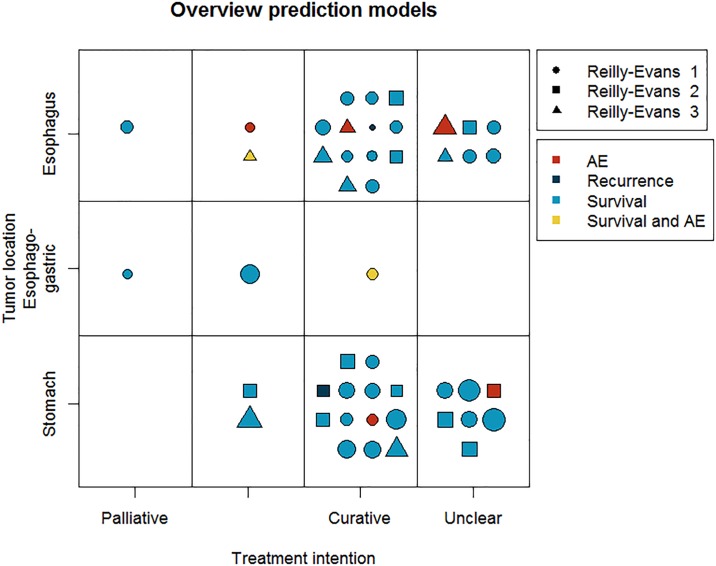
Overview of included prediction models. The shape indicates the type of study and the size of shapes indicate the pooled c-index. Larger sizes of shapes indicate higher c-indices. AE = adverse event; Reilly-Evans = levels of evidence on the discriminatory accuracy of the prediction model described by Reilly and Evans[14], which indicate how extensively a prediction model has been validated and to what extent a model is ready for clinical use.

Of the models described in the 45 development studies, six predict adverse events; one predicts the recurrence of malignancy; and most studies (N = 39) predict various types of survival (six disease-free survival, eight disease-specific survival, 23 overall survival and five post-operative mortality). None of the studies predict HRQoL and none predict more than one outcome, i.e., no model predicts both the harms and benefits of the treatments of interest. The majority of studies (N = 28) used a nomogram to present the prediction model, while others (N = 13) used a formula as a presentation method (see Table 1). Three prediction models were also available online. A graphical overview of the outcomes per prediction model is given in Fig 2, and includes depiction of each model’s Reilly-Evans level of evidence on discriminatory accuracy.

Table 3 provides an overview of the selected studies. Most models underwent only limited validation, as the majority of development models were not validated further in later studies. This is expressed by the Reilly and Evans levels of evidence[14]. In 84% of the development studies the two lowest Reilly and Evans levels, namely 1 or 2, were scored indicating only narrow validation. The validation studies are limited to a select group of prediction models, which are validated more extensively. These are the prediction models developed by Eom 2015[30], Lagarde 2007[44], Lagarde 2008[45], Lai 2009[46], Marelli 2005[50], Steyerberg 2006[57], the MSKCC[83], and the Possum[19], O-Possum[60], and P-Possum[20] models. This more extensive validation resulted in a majority of these models having a Reilly and Evans level of 3.

**Table 3:** Overview of study characteristics in development and validation studies.

	Development studies	Validation studies	P-value
**N**	45	16	
**Reilly-Evans level (%)**			
- 1	27 (60.0)	0 (0.0)	
- 2	11 (24.4)	3 (18.8)	
- 3	7 (15.6)	13 (81.2)	
**Continent of patient population (%)**			p = 0.003
- Asia	25 (56.8)	3 (18.8)	
- Europe	8 (18.2)	11 (68.8)	
- North-America	10 (22.7)	2 (12.5)	
- North-America and Europe	1 (2.3)	0 (0.0)	
**Intended time of model use (%)**			p = 0.857
- After adjuvant chemotherapy	1 (2.2)	0 (0.0)	
- After consolidation therapy	1 (2.2)	0 (0.0)	
- After definitive chemoradiation	1 (2.2)	0 (0.0)	
- After resection	32 (71.1)	14 (87.5)	
- At diagnosis	1 (2.2)	0 (0.0)	
- Before definitive chemotherapy	1 (2.2)	0 (0.0)	
- Before resection	5 (11.1)	2 (12.5)	
- Before/after resection	3 (6.7)	0 (0.0)	
**Curative/palliative setting (%)**			p = 0.316
- Curative	25 (55.6)	13 (81.2)	
- Curative/Palliative	5 (11.1)	1 (6.2)	
- Palliative	2 (4.4)	0 (0.0)	
- Unclear	13 (28.9)	2 (12.5)	
**Calibration method (%)**			p = 0.045
- Calibration plot	23 (51.1)	6 (37.5)	
- Statistical analysis	2 (4.4)	4 (25.0)	
- Calibration plot and statistical analysis	6 (13.3)	4 (25.0)	
- None	14 (31.1)	2 (12.5)	

Table 3 also indicates the study patient distribution across the continents. This differs significantly between development and validation studies (p = 0.003), indicating that different populations are used for model development and for validation. This difference is especially pronounced between Asia and Europe (p < 0.001). Models were more often developed in Asian than in European populations (56.8% vs. 18.2% respectively), however, fewer validation studies were conducted in Asian than in European populations (18.8% vs 68.8% respectively). The development and validation studies mostly concerned prediction outcomes before or after resection (89% and 100% respectively), and were mostly aimed at patients treated with curative intent (56% and 81.2% respectively).

### Bias analyses

We analyzed several areas of possible bias of the studies, which are shown in Tables 4 and 5. The exact definitions of the biases are presented in S2 Table. Of all selected studies, population-related bias occurred in 61%, predictor-related bias in 43%, outcome-related bias in 43%, sample size -related bias in 38%, missing data-related bias in 89% and statistical analysis-related bias in 66%. All studies have a bias in at least one area. Due to poor or inconsistent reporting, it was difficult to extract pertinent study information. For example, treatment intent was not reported in most articles. In such cases intent was deduced from other available information such as the presence of metastatic disease. However, in fifteen studies the treatment intent could not be established. Also, unclear descriptions of treatment and patient characteristics limited our ability to evaluate the risk of bias. The potential source of bias that was most difficult to evaluate due to poor reporting, concerns the handling of missing data. Although few studies report that their dataset was complete, most studies did not mention whether this was the case and how they handled missing data (e.g., via multiple imputation). Further, in many studies, it was unclear what outcome was being predicted. For example, authors mention ‘survival’ as an outcome[51], but it remained unclear whether overall survival or disease-specific survival was implied.

**Table 4:** Overview of areas of bias in the included studies (part 1).

	Subject bias	Predictor bias	Outcome bias	Sample size bias	Missing data bias	Statistical analysis bias
Ashfaq 2015[67]	-	+	+	++	--	-
Biglarian 2011[22]	-	-	?	-	--	--
Bosch 2011[68]	+	+	-	-	-	-
Cao 2016[23]	-	+	?	+	-	+
Chen, D. 2013[69]	+	+	+	+	--	-
Chen, S. 2016[24]	--	+	+	-	-	-
D’Journo 2016[70]	--	+	+	+	-	+
Deans 2007[25]	+	-	+	-	-	-
Dhir 2012[26]	-	-	?	++	-	+
Dikken 2013[27]	-	-	+	+	--	-
Dikken 2014[71]	-	+	-	-	--	-
Duan 2016[28]	+	+	-	+	--	+
Eil 2014[29]	--	-	--	+	--	-
Eom 2015[30]	-	-	+	-	--	+
Filip 2015[31]	--	-	--	-	-	-
Fischer 2016[32]	+	-	+	+	+	-
Fuccio 2016[33]	-	+	--	-	--	-
Gabriel 2017[34]	-	-	+	++	--	-
Grotenhuis 2010[72]	+	-	-	+	+	-
Haga 2015[35]	-	+	--	+	-	-
Han 2012[36]	-	+	?	+	--	+
Hirabayashi 2014[37]	+	+	?	+	--	-
Jiang 2016[38]	--	+	--	-	-	-
Jung 2013[39]	-	+	-	-	-	+
Kattan 2003[40]	-	-	+	+	--	-
Kim, J.H. 2012[73]	+	-	-	+	--	-
Kim, Y. 2015[41]	+	+	--	+	-	-
Kunisaki 2016[42]	-	+	-	++	-	+
Kurita 2015[43]	+	-	-	+	-	-
Lagarde 2007a[74]	+	+	-	+	-	-
Lagarde 2007b[44]	--	+	+	+	?	-
Lagarde 2008a[45]	+	+	-	+	+	-
Lagarde 2008b[75]	+	-	+	-	+	-
Lai 2009[46]	+	-	+	+	--	-
Liu, J. 2016a[47]	-	+	?	+	-	+
Liu, J. 2016b[48]	+	-	?	+	--	+

A minus sign indicates possible areas of bias; a question mark indicates that bias could not be determine;. a positive sign indicates a lack of bias.

**Table 5:** Overview of areas of bias in the included studies (part 2).

	Subject bias	Predictor bias	Outcome bias	Sample size bias	Missing data bias	Statistical analysis bias
Liu, J.S. 2015[49]	--	-	-	-	-	-
Marrelli 2005[50]	+	-	?	-	-	-
Marrelli 2015[76]	+	+	+	+	--	+
Mohammadzadeh 2015[51]	--	-	--	-	--	--
Muneoka 2016[52]	-	+	-	-	-	-
Nagabhushan 2007[77]	+	+	-	-	--	-
Novotny 2006[78]	-	+	+	+	--	-
Peeters 2005[79]	-	-	+	-	--	-
Reim 2015[80]	--	+	+	+	+	+
Shao 2015[53]	+	+	+	+	-	+
Shapiro 2016[54]	+	-	-	+	-	-
Shiozaki 2016[55]	+	+	+	--	--	-
Song 2014[56]	-	-	--	+	--	+
Steyerberg 2006[57]	-	-	+	+	?	-
Su 2015[58]	--	+	+	+	--	+
Suzuki 2012[59]	--	+	?	-	-	-
Tekkis 2004[60]	+	-	+	+	-	-
Tu 2017[61]	+	+	--	+	--	-
Woo 2016[62]	-	+	?	++	-	+
Yang 2013[63]	-	+	+	-	-	-
Yu 2016[64]	+	+	+	+	-	+
Zafirellis 2002[81]	-	+	--	-	--	+
Zhao 2016[65]	-	-	--	-	-	+
Zhou, M.L. 2016[82]	-	+	+	-	--	+
Zhou, Z. 2015[66]	-	-	-	+	-	+

A minus sign indicates possible areas of bias; a question mark indicates that bias could not be determine;. a positive sign indicates a lack of bias.

In most studies the model calibration was poorly reported. Although 45 out of 61 studies described some form of calibration, only 16 studies performed a formal statistical calibration analysis to support whether the predicted risk matched the observed risk. None of the studies determined the calibration slope and intercept (which represents the systematic over- or underprediction of risk).

Finally, we also investigated whether the impact factor of the journal in which the study was published influenced the amount of bias. We found no significant correlation between journal impact factor and the risk of population-related bias (rho = 0.09, p = 0.51), predictor-related bias (rho = -0.12, p = 0.37), outcome-related bias (rho = 0.17, p = 0.20), sample size-related bias (rho = 0.13, p = 0.32), missing data-related bias (rho = 0.03, p = 0.79) or statistical analysis-related bias (rho = 0.03, p = 0.80). When we assessed whether models published in high impact journals performed better in terms of discriminative ability, again, we found no relation between the impact factor of the journal and the reported c-index (rho = 0.15, p = 0.11).

### Meta analyses of c-indices

**Fig 3:**
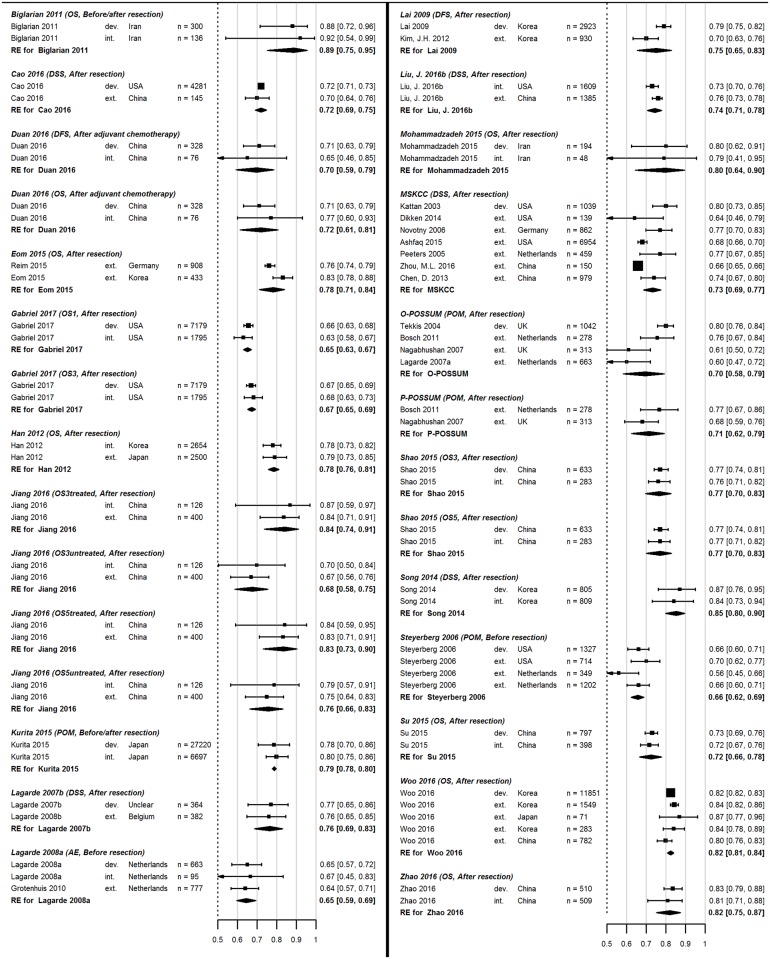
Random effects meta analyses of the discriminative abilities (c-indices) of the identified prediction models. DSS: disease-specific survival, POM: post-operative mortality, OS: overall survival, AE: adverse events, DFS: disease-free survival, REC: cancer recurrence, dev: development c-index, int: internal validation, ext: external validation.

Results of the meta-analysis of available c-indices of corresponding prediction models are shown in Fig 3. Results are pooled per prediction model and are indicated by diamonds. Overall, the meta-analysis highlights that there is great uncertainty about the predictive performances of available models, given the large confidence intervals (with ranges >0.1) in most pooled estimates. Furthermore, the pooled estimates show that the models vary in discriminating ability, ranging from 0.65 (poor discrimination) to 0.85 (good discrimination), with an average pooled estimate of 0.75 (fair discrimination).

To investigate whether model overfitting occurs, that is the discriminative ability of a model is overestimated during training, we examined the difference in model c-indices. It was found that the discriminative ability of the model was indeed larger (p = 0.01) in development (average c-index: 0.76) than in validation studies (average c-index = 0.73).

## Discussion

The main aim of this review was to provide an overview of prediction models aimed at predicting survival, adverse events and HRQoL in patients with esophageal or gastric cancer, and establish their predictive performance and biases.

We identified 45 articles describing the development of novel prediction models and only 16 studies validating these prediction models. We were unable to perform meta-analyses of model calibration, as studies either did not or not adequately report model calibration. The meta-analyses of model discriminative abilities indicate large heterogeneity. The pooled estimates of the discriminative abilities tended to have large confidence intervals, which can be explained by low levels of validation and small cohort sizes. The identified studies generally report a fair discriminative ability for the prediction models. Although nearly every study states that the model is potentially useful in practice, almost all studies do acknowledge the need for further external model validation. However, a mere 10 out of 47 prediction models were subsequently tested in such external validation studies. Indeed, the importance of external validation is shown by the present study as we found that the discriminative ability of models was significantly lower in the validation than in the development phase. Presenting only development results may lead to optimism bias and should be acknowledged when using the prediction models in clinical practice. Large datasets are increasingly being made (freely) available online, which may facilitate more extensive validation of prediction models in the future.

Our findings highlight that prior to using any of these prediction models in clinical practice, clinicians need to carefully consider the number and quality of available validations, the countries/populations in which the models were validated, sample sizes and study biases. In fact, the reported low Reilly and Evans levels of validation indicate that the models we have identified are not ready for widespread implementation in clinical practice. Despite the absence of clinically relevant models, the reported results are essential for future benchmarking and validation studies. Eight models have reached a Reilly and Evans level 3, with the MSKCC model being the most promising with a pooled c-index of 0.73, and extensive validation in a wide-range of populations and settings. We recommend that the MSKCC will be further investigated for its added value in clinical practice in terms of, for example, reduction of decisional conflict and increased patient participation (i.e., shared decision making). Only when the quality of care is improved following implementation of the model, its widespread use in clinical practice can be recommended.

Most of the identified models focus on prediction of survival after curative resection of esophageal or gastric cancer. Although these models provide insight into prognosis of this particular group of patients, they are of limited value for treatment decisions, as treatment has largely been completed at the point of resection. Furthermore, none of the prediction models predict HRQoL, despite the established relevance of HRQoL when making treatment decisions[7], especially in the palliative setting. Finally, in order to make a well-informed treatment choice, patients need to consider both the benefits and harms of treatments to determine which option best fits their preferences and goals. However, none of the prediction models we identified provide estimates of both the benefits and harms associated with a treatment option. Thus, if clinicians opt to use the currently available models, it is imperative that they supplement the information provided by the model with evidence-based predictions concerning not only the possible increase in life-span, but also the possible adverse events and impact on HRQoL.

In order to assess the quality of the studies, we determined sources of possible bias in six different areas. Most studies had a high risk of bias, and all articles showed possible bias in at least one area. The most common bias concerned the handling of missing data. In many studies, it was unclear whether data was missing, how much was missing and how the missing data were handled. Model calibration was not mentioned in some cases and often not accompanied by statistics to provide insight into model quality. Overall, the quality of reporting was poor. Crucial information needed for the interpretation of the results was ill-reported, such as when the model should be used, if the model was to be used with patients for whom treatment has a palliative or curative intent, and what the confidence intervals of the outcomes were. We did not contact authors in cases where the reporting was incomplete, as the focus of this study was to create an overview of reported studies and not to analyze bias in prediction models per se. We strongly advocate that when reporting the development or validation of prediction models the guidance in TRIPOD-statement[15] is followed. This statement provides a checklist of necessary items to include when reporting prediction model development and validation studies, which would facilitate a consistent manner of reporting and safeguard the inclusion of important items needed for interpretation of the data.

In contrast to our expectation, we found no relation between the predictive performance of the models and the impact factor of the journal in which the study is published, nor between the impact factor and study bias. Clinicians should keep in mind that a high impact factor is not a guarantee for quality, and they should always critically assess the quality and generalizability of the prediction model for use in clinical practice. The results of the current study may aid such an evaluation.

In conclusion, we found 47 prediction models intended to predict outcomes in patients with esophageal and gastric cancer. Most models mainly aimed to predict survival after curative resection. Validation of these models is generally limited and the overall performance was fair. There is a clear need for new prediction models for patients with esophageal and gastric cancer that focus on both the potential benefits (e.g., improved survival) and harms (e.g., occurrence of adverse events and/or loss of quality of life) of treatment. Such comprehensive prediction models will likely support the decision-making process.

## Supporting information

S1 Table Search strategy per database. (DOC)

S2 Table Overview and categorization of potential sources of bias identified in included articles. (DOC)

S3 Table PRISMA[21] checklist. (DOC)
