# Supplementary material for: Prediction models for patients with esophageal or gastric cancer: A systematic review and meta-analysis
Source: PLoS One. 2018 Feb 8;13(2):e0192310. doi: 10.1371/journal.pone.0192310 (PMC5805284; doi:10.1371/journal.pone.0192310)
Supplement: S1 Table — (DOC) [file pone.0192310.s001.doc]

| ***Database*** | ***Search terms*** |
| --- | --- |
| **MEDLINE** | (((*Esophageal Neoplasms/ or *Stomach Neoplasms/ or ((esophagus or esophageal or esophagogastric or oesophagus or oesophageal or oesophagogastric or gastroesophag* or gastrooesophag* or gastric or stomach) adj (neoplas* or cancer* or carcino* or adenocarcino* or tumor* or tumour* or malig*)).ti,ab,kw. AND Survival/ or exp Survival Analysis/ or Survival Rate/ or Neoplasm Metastasis/ or Neoplasm Recurrence, Local/ or Comorbidity/ or "Quality of Life"/ or exp Mortality/ or Life Expectancy/ or (surviv* or mortalit* or toxic* or disease free survival or quality of life or QOL or life quality or recurrence or metastas* or comorbidity or life expectanc* or adverse effect* or adverse event*).ti,ab,kw. AND (rule* or scor* or model* or nomogram* or regression* or network* or predict*).ti,ab,kw. AND exp models, statistical/ or exp Regression Analysis/ or Prognosis/ or (validat* or prognos* or predict*).ti,ab,kw.) AND (academic dissertations/ or classical article/ or journal article/ or introductory journal article/ or "review"/)) NOT Animals/ not Humans/) limit to (english language and yr="2000 - 2017") |
| **EMBASE** | ((exp *esophagus tumor/ or exp *stomach tumor/ or ((esophagus or esophageal or esophagogastric or oesophagus or oesophageal or oesophagogastric or gastroesophag* or gastrooesophag* or gastric or stomach) adj (neoplas* or cancer* or carcino* or adenocarcino* or tumor* or tumour* or malig*)).ti,ab,kw. AND exp survival/ or survival analysis/ or survival rate/ or metastasis/ or tumor recurrence/ or cancer recurrence/ or comorbidity/ or "quality of life"/ or exp mortality/ or life expectancy/ or (surviv* or mortalit* or toxic* or quality of life or QOL or life quality or recurren* or metastas* or comorbidit* or life expectanc* or adverse effect* or adverse event*).ti,ab,kw. AND (rule* or scor* or model* or nomogram* or regression* or network* or predict*).ti,ab,kw. AND statistical model/ or exp regression analysis/ or exp prognosis/ or (validat* or prognos* or predict*).ti,ab,kw.) AND (scientific literature/ or article/ or "review"/)) NOT (animal/ not human/) limit to (english language and yr="2000 - 2017") |
| **PsycINFO** | (((esophagus/ or exp Gastrointestinal Disorders/ or exp Gastrointestinal System/ or exp Stomach/) and exp Neoplasms/) or ((esophagus or esophageal or esophagogastric or oesophagus or oesophageal or oesophagogastric or gastroesophag* or gastrooesophag* or gastric or stomach) and (neoplas* or cancer* or carcino* or adenocarcino* or tumor* or tumour* or malig*)).ti,ab,id. AND mortality rate/ or metastasis/ or comorbidity/ or "quality of life"/ or "death and dying"/ or life expectancy/ or (surviv* or mortalit* or toxic* or quality of life or QOL or life quality or recurren* or metastas* or comorbidit* or life expectanc* or adverse effect* or adverse event*).ti,ab,id. AND (rule* or scor* or model* or nomogram* or regression* or network* or predict*).ti,ab,id. AND exp mathematical modeling/ or exp statistical regression/ or prognosis/ or (validat* or prognos* or predict*).ti,ab,id.) limit to human AND (english language and yr="2000 - 2017") |
| **CINAHL** | (MH "Esophageal Neoplasms") OR (MH "Stomach Neoplasms") OR (TI ( (esophagus or esophageal or esophagogastric or oesophagus or oesophageal or oesophagogastric or gastroesophag* or gastrooesophag* or gastric or stomach) N1 (neoplas* or cancer* or carcino* or adenocarcino* or tumor* or tumour* or malig*)) OR AB ( (esophagus or esophageal or esophagogastric or oesophagus or oesophageal or oesophagogastric or gastroesophag* or gastrooesophag* or gastric or stomach) N1 (neoplas* or cancer* or carcino* or adenocarcino* or tumor* or tumour* or malig*) ) ) AND ((MH "Survival") OR (MH "Survival Analysis+") ) OR (MH "Neoplasm Metastasis+") OR (MH "Neoplasm Recurrence, Local") OR (MH "Comorbidity") OR (MH "Quality of Life+") OR (MH "Mortality+") OR (MH "Life Expectancy") OR ( TI ( surviv* or mortalit* or toxic* or quality of life or QOL or life quality or recurren* or metastas* or comorbidit* or life expectanc* or adverse effect* or adverse event* ) OR AB ( surviv* or mortalit* or toxic* or quality of life or QOL or life quality or recurren* or metastas* or comorbidit* or life expectanc* or adverse effect* or adverse event* )) AND TI ( rule* or scor* or model* or nomogram* or regression* or network* or predict*) OR AB ( rule* or scor* or model* or nomogram* or regression* or network* or predict*) AND  (MH "Models, Statistical") OR (MH "Regression+") OR (MH "Prognosis+") OR (TI ( validat* or prognos* or predict* ) OR AB ( validat* or prognos* or predict* )) |
| **The Cochrane Library** | (esophagus or esophageal or esophagogastric or oesophagus or oesophageal or oesophagogastric or gastroesophag* or gastrooesophag* or gastric or stomach) and (neoplas* or cancer* or carcino* or adenocarcino* or tumor* or tumour* or malig*):ti,ab,kw  (Word variations have been searched) AND surviv* or mortalit* or toxic* or quality of life or QOL or life quality or recurrence or metastas* or comorbidity or life expectanc* or adverse effect* or adverse event*:ti,ab,kw  (Word variations have been searched) AND rule* or scor* or model* or nomogram* or regression* or network* or predict*:ti,ab,kw  (Word variations have been searched) AND (MeSH descriptor: [Models, Statistical] explode all trees OR MeSH descriptor: [Regression Analysis] explode all trees OR MeSH descriptor: [Prognosis] explode all trees OR validat* or prognos* or predict*:ti,ab,kw  (Word variations have been searched)) Publication Year from 2000 to 2017 |
