# Supplementary material for: Prediction models for patients with esophageal or gastric cancer: A systematic review and meta-analysis
Source: PLoS One. 2018 Feb 8;13(2):e0192310. doi: 10.1371/journal.pone.0192310 (PMC5805284; doi:10.1371/journal.pone.0192310)
Supplement: S2 Table — (DOC) [file pone.0192310.s002.doc]

| **Study participant** |  |
| --- | --- |
| **++** | - Prospective study |
| **+** | - No potential source of bias related to study participants identified - High case-mix |
| **?** | None identified |
| **-** | - Selection bias - Treatment out of date - Setting not clearly described - Not clearly described whether patients received adjuvant treatment - Limited generalizability |
| **--** | - Unclear what main treatment modality was - Unclear description in- and exclusion criteria - Censoring bias - Patient characteristics not described |
| **Predictors** |  |
| **++** | None identified |
| **+** | - No potential source of bias related to predictors identified |
| **?** | None identified |
| **-** | - Arbitrary cut-off utilized - Arbitrary predictor selection - Relevant predictors not considered - Predictors not defined |
| **--** | None identified |
| **Outcome** |  |
| **++** | None identified |
| **+** | - No potential source of bias related to outcome identified |
| **?** | - Outcome not clearly specified - Number of events not reported |
| **-** | - Degrees of Freedom/events ratio < 10 in development set - Timing short-term outcome (e.g., post-operative mortality) unclear |
| **--** | - Unclear what main outcome is - Timing long-term outcome unclear |

| **Sample size** |  |
| --- | --- |
| ++ | - More than 5000 cases included in analyses |
| + | - No potential source of bias related to sample size identified - >550 and <5000 cases |
| ? | None identified |
| - | - Small sample size (fewer than 550 cases) - Unsuitable imputation technique |
| -- | - Fewer than 100 cases - Imputation by reference category in nominal variable |
| **Missing data handling** |  |
| ++ | None identified |
| + | - No potential source of bias related to missing data handling identified - Multiple imputation |
| ? | - Unclear which imputation technique was used |
| - | - Unclear how missing data was handled |
| -- | - Complete case analyses |
| **Statistical analyses** |  |
| ++ | None identified |
| + | - No potential source of bias related to the statistical analyses identified |
| ? | None identified |
| - | - Logistic regression for survival (except if outcome was post-operative mortality) - Confidence intervals were not reported - Calibration was not assessed |
| -- | - Discriminatory ability: area under the curve (AUC) nor C-index reported |
